# Assessing healthy distrust in human-AI interaction: interpreting changes in visual attention

**DOI:** 10.3389/fpsyg.2025.1694367

**Published:** 2026-01-02

**Authors:** Tobias M. Peters, Kai Biermeier, Ingrid Scharlau

**Affiliations:** Department of Psychology, Faculty of Arts and Humanities, Paderborn University, Paderborn, Germany

**Keywords:** appropriate trust, healthy distrust, visual attention, Theory of Visual Attention, human-AI interaction, Bayesian cognitive model, image classification

## Abstract

**Introduction:**

When humans interact with artificial intelligence (AI), one desideratum is appropriate trust. Typically, appropriate trust encompasses that humans trust AI except for instances in which they either explicitly notice AI errors or are suspicious that errors could be present. So far, appropriate trust or related notions have mainly been investigated by assessing trust and reliance. In this contribution, we argue that these assessments are insufficient to measure the complex aim of appropriate trust and the related notion of healthy distrust. We introduce and test the perspective of covert visual attention as an additional indicator for appropriate trust and draw conceptual connections to the notion of healthy distrust.

**Methods:**

To test the validity of our conceptualization, we formalize visual attention using the Theory of Visual Attention and measure its properties that are potentially relevant to appropriate trust and healthy distrust in an image classification task. Based on temporal-order judgment performance, we estimate participants' attentional capacity and attentional weight toward correct and incorrect mock-up AI classifications. We observe that misclassifications reduce attentional capacity compared to correct classifications. However, our results do not indicate that this reduction is beneficial for a subsequent judgment of the classifications.

**Results and discussion:**

The attentional weighting is not affected by the classifications' correctness but by the difficulty of categorizing the stimuli themselves. We discuss these results, their implications, and the limited potential for using visual attention as an indicator of appropriate trust and healthy distrust.

## 1 Introduction

In current research on human-AI interaction, a common desideratum is appropriate trust (Kastner et al., 2021; Mehrotra et al., 2024; Visser et al., 2025). Appropriate trust entails that a person would trust AI when it is correct or suitable for a specific task, but not when it is incorrect or unsuitable for that task. Given that the actual capabilities of AI are imperfect, appropriate trust and related concepts are a much-needed improvement over simply aiming to foster trust. However, approaches investigating if and how these imperfect capabilities are noticed, and what consequences they have on users, are limited.

While a unified definition does not yet exist, appropriate trust and similar concepts all revolve around the aim to align the perceived and the actual capabilities of AI (Mehrotra et al., 2024). This alignment can be defined as appropriate reliance (Lee and See, 2004; Peters and Visser, 2023). This notion does, however, entail problematic issues. Before focusing on them, let us first define the relevant elements. Reliance can be defined as a decision or action that takes AI support into account (Visser et al., 2025). Trust “plays a leading role in determining the willingness of humans to rely on automated systems in situations characterized by uncertainty” (Hoff and Bashir, 2015, p. 407). Furthermore, two types of reliance problems can occur: Overtrust occurs when one relies on AI even when it is incorrect. Disuse occurs when one does not rely on AI even when it is correct. For appropriate reliance, both problems should be prevented. Thus, theoretically, appropriate reliance would be achieved if a person always relies when the AI support is correct and never relies when it is incorrect.

Problematically, it is questionable whether a person with the necessary knowledge for this would need AI support in the first place. Moreover, a situation in which someone can rely appropriately all the time would not be characterized by uncertainty. In terms of the connection to trust, this is problematic because trust is only of conceptual relevance when the outcome is uncertain (see definition above, and Mayer et al., 1995; Mühlfried, 2018). While we can imagine that there are some applications where one can always identify the AI's correctness, we think that those applications where this surpasses human knowledge are the ones of interest for (envisioned) AI use cases. In these cases, we regard achieving full appropriate reliance more as a theoretical aim, given the uncertainty of relying on such AI applications. Instead of leading to appropriate reliance, appropriate trust and related concepts aim to improve appropriate reliance. Furthermore, depending on the context, the risk of relying on AI may differ, which in turn decides whether overtrust, disuse, or both equally are more detrimental. Thereby, when and how much trust is appropriate varies and can, thus, change for each context.

To summarize, appropriate reliance is what appropriate trust aims to achieve. The ideal state with which appropriate reliance is described oversimplifies the problem it tackles. Not always knowing the ground truth is a key characteristic of scenarios where AI is beneficial. This is also reflected in descriptions of ideal human-AI interactions. Such descriptions often refrain from stating that users should be able to identify a model's errors. Instead, users should “form a correct mental model of the model's error boundaries” because this would allow them to know when to trust or distrust the model's recommendations (Zhang et al., 2020, p. 295). Alternatively, they should be enabled to differentiate when the reasoning of an AI is correct and when suspicion would be warranted (Bansal et al., 2021). These descriptions exemplify the range of desirable consequences when faced with incorrect or unsuitable AI support. Beyond explicitly noticing errors, these consequences also include a sense of when to be suspicious. Thus, the aim of appropriate trust might best be described as a combination of an explicit and conscious identification of errors and a potentially unconscious intuition of when to doubt, which parallels the work on epistemic vigilance (Sperber et al., 2010). Conceptualizing and assessing such a range of consequences strikes us as both necessary and demanding.

So far, the notion of appropriate trust has been mostly investigated through the concepts of trust and reliance. Reliance is the decision made after seeing the AI output and potentially descriptors such as the input, model information, or explanations. Trust, typically assessed via self-report, is a user's introspective account that may be, and often is, influenced by errors (e.g., Yu et al., 2018; Yin et al., 2019; Peters and Scharlau, 2025). However, it is also influenced by a variety of other factors, including pre-existing knowledge, personality traits, and situational factors (Hoff and Bashir, 2015; Kaplan et al., 2023). For reliance, the common result is also that it decreases in the presence of errors (e.g., Yin et al., 2019; Papenmeier et al., 2022). Notably, discrepancies between trust and reliance have been reported (Papenmeier et al., 2022; Lammert et al., 2024), which emphasize the need to investigate both because people may rely even though they report little trust or do not rely despite reporting trust. For the present argumentation, it is especially important to point out two aspects: (i) trust and, thereby, reliance are influenced by many factors besides the actual trustworthiness, and (ii) they do not directly indicate whether people notice potential errors and how they may be affected by them.

The latter is problematic because the results obtained from these established operationalizations leave room for different causal pathways and influences on trust and reliance. For example, Lai and Tan (2019) report that, in general, their participants trusted correct advice more than incorrect advice. They also observed, however, that trust was increased by adding randomly generated heatmaps as explanations, which did not justify an increase in trust. It remains unresolved whether their participants did not notice that the recommendations were incorrect or if they did notice but relied on them anyway.

One way to overcome such limitations would be to let participants indicate their perception of the recommendation's correctness. However, as we discussed above, appropriate trust and similar concepts are not only about clearly noticing errors but also about merely sensing possible errors. The latter may be difficult to explicate and thus also difficult to operationalize via self-report. Therefore, extending the typical assessments of self-report and reliance seems advisable for a more thorough analysis of human-AI interactions. In this contribution, we want to introduce the perspective of visual attention as one important aspect influencing the initial perception of and subsequent interaction with AI. Thereby, we want to suggest and test another indicator of appropriate trust, or rather, healthy distrust, and thereto also highlight conceptual issues of trust and distrust.

Readers may deem it far-fetched to suggest visual attention as an indicator of appropriate trust or similar concepts. However, visual attention covers early processes involved in information processing within human-AI interaction, and it is a core function relevant for the selection and recognition of visual stimuli (Bundesen, 1990; Evans et al., 2011). Accordingly, we consider visual attention highly relevant in detecting (potential) errors in visual scenarios. For example, consider a medical professional viewing a mammography image of a dense breast classified as cancerous. Given the professional's knowledge that, for such cases, mammography is a poor choice of imaging technique (Calisto et al., 2021), this classification would seem wrong to them. Thus, the image may receive more attention than an image that does not indicate such an error.

Moreover, methods from visual attention research are well-equipped to quantify such changes in attentional weighting, i.e., the distribution changes of the overall attentional capacity (Bundesen et al., 2015). Therefore, measuring a person's attention while interacting with erroneous AI may shed further light on whether and how people notice errors (consciously or unconsciously), and ultimately, may provide an indicator for (dis)trust and thus be a means to improve the assessment of appropriate trust and healthy distrust. We will describe this in more detail in the following.

### 1.1 Healthy distrust & visual attention

Recently, we and others (Kohn et al., 2021; Peters and Visser, 2023; Paaßen et al., 2025) have advocated for not considering only trust but also distrust in the current AI context. This is based on at least three issues: i) the conceptualization of trust and distrust as two related yet separate dimensions, ii) the benefits of distrust, and iii) the current focus on trust in the AI context. Firstly, Lewicki et al. (1998) prominently proposed trust and distrust as two related yet separate dimensions. Unfortunately, this theoretically and empirically well-justified suggestion had received little attention, neither in Psychology nor in related disciplines (Vaske, 2016). This is also true for the context of AI, even though a few recent exceptions exist (Kohn et al., 2021; Colville and Ostern, 2024; Scharowski et al., 2025). What is promising about this two-dimensional approach is that it conceptually allows for trusting and distrusting something at the same time. This state may be an element of vigilance or suspicion, which might turn out to be elements of appropriate trust. This co-existence can be difficult to imagine, but especially for measuring appropriate trust, it may be important to assess trust and distrust separately.

Despite the typical negative connotation, distrust can also have beneficial consequences, such as increasing creativity or memory performance (Mayer and Mussweiler, 2011; Posten and Gino, 2021) and reducing confirmatory biases (Mayo, 2015). These are benefits that would contribute to the aim of appropriate trust and appropriate reliance. Typical characteristics of distrust are skepticism, vigilance, and wariness (Lewicki et al., 1998; Cho, 2006). Following the two-dimensional conceptualization of trust and distrust, only evaluating trust would neglect these interesting characteristics that are likely to be crucial for detecting errors or the potential for them, and that are, thus, important when AI's actual capabilities are poor.

However, only 14% of studies on appropriate trust also consider and measure distrust (Mehrotra et al., 2024). As recently argued, this could be improved by aiming to foster a healthy distrust alongside appropriate trust (Paaßen et al., 2025). Healthy distrust can be understood as an expression of “a careful or negative stance” (Paaßen et al., 2025, p.15) and is distinct from instilling outright distrust. This distinction is important because, as Mayo (2024) argues, distrust is a “double-edged sword” and can, just like trust, lead to unwanted consequences such as an increase in conspiracy beliefs and a neglect of correct information. Accordingly, she suggests fostering an evaluative mindset rather than a distrust mindset. The evaluative mindset entails making a pause instead of directly accepting or rejecting information.

Because vigilance and wariness are closely connected to attention, we suggest the assessment of attention as a promising indicator for healthy distrust and a useful addition to the assessment of self-reported (dis)trust and reliance. Given our experimental scenarios, we will focus on visual attention in the following. As a starting point, we will manipulate the presence and absence of errors via mock-up AI classifications, and we will focus on single-turn decisions. This simplifies what appropriate trust and healthy distrust encompass. As discussed above, these notions also apply to the question of whether a certain model is trusted or distrusted for a specific task, and not only to such single-turn interactions. However, as a first step, this simplification is useful.

To measure visual attention, we instantiate the Theory of Visual Attention (TVA; Bundesen, 1990) to describe task performance in a specific experimental paradigm (Tünnermann et al., 2015). This allows us to quantify the overall attentional capacity *C* and attentional weights *w* that certain stimuli receive. Both parameters have the potential to serve as an indicator of distrust. For instance, wrongly classified images that are distrusted may attract more attention, which would be reflected in *w*. Moreover, distrust caused by the incongruency between image type and its classification in the case of misclassification may also affect the overall attentional capacity. It could lead to a higher *C*, indicative of a more attentive state, or to a lower *C*, indicative of attentional detriments due to additional demands of such incongruencies.

TVA formalizes visual attention as a fixed-capacity independent race. This means that a fixed capacity is spread across all stimuli within the visual field. Each of the stimuli races for representation in visual short-term memory (Bundesen et al., 2015). Each stimulus has a processing speed *v*, which determines how likely it is that the stimulus wins the race for representation in visual short-term memory. The processing speed of a stimulus depends on its visual evidence, a decision bias, and, central for this contribution, on its attentional weight *w* (for further details, see Section 2.5.1 and e.g., Bundesen et al., 2015; Tünnermann et al., 2017). The attentional weight determines how much of the attentional capacity the stimulus receives. If a stimulus has a higher *w*, it is more likely to finish the race first.

Following Tünnermann et al. (2015, 2017), we use temporal-order judgments (TOJ). The TVA-TOJ approach has the advantage that it consists of a task that is easily combined with our stimulus material, or, for that matter, with practically any visual stimulus material (Krüger et al., 2021).

### 1.2 Research questions & hypotheses

In this paper, we investigate how decision difficulty and AI errors affect visual attention as a potential indicator of (healthy) distrust and test if participants' task performance is improved by an option to deliberate on their judgment. In the present study, the AI errors are incorrect image classifications. To assess visual attention, we investigate the overall attentional capacity *C* that participants have in our experimental scenario and the attentional weight *w* that certain types of stimuli receive. Furthermore, we study whether the option to withhold a decision by choosing that the image and its classification are shown again later (SAL-option) improves the combined human-AI performance compared to two alternative forced-choice decisions (2-AFC). Based on this, we investigate the following research questions:

- RQ1: Do images that are more difficult to categorize receive a higher attentional weight *w* than more easily categorizable images?
- RQ2: Do images classified wrongly lead to a different attentional capacity *C* than correctly classified images?
- RQ3: Do wrongly classified images receive a higher attentional weight *w* than correctly classified images?
- RQ4: Does the SAL-response option improve performance compared to the 2-AFC decisions?

Research Question 1 is investigated in Experiment 1, while the other research questions are investigated in Experiment 2. After the participants are familiarized with the material, we assess whether images that are more difficult to categorize receive more attention. Thus, as *Hypothesis 1*, we expect a higher attentional weight *w* for images that are difficult to classify than for images that are easy to classify. To address Research Question 2, we compare the *C* estimates of trials with two misclassifications to those of trials with two correct classifications. As *Hypothesis 2*, we expect a difference between these two trial types. Research Question 3 will be studied using trials with one correct and one incorrect classification. Because we assume increased attention toward potentially incorrect decisions, we expect a higher attentional weight *w* for the incorrect classifications as *Hypothesis 3*. For Research Question 4, we compare the participants' performance when making 2-AFC decisions and when using the SAL-option. As *Hypothesis 4*, we expect higher performance in the trials with the SAL-option.

## 2 Methods

To investigate the research questions and test the hypotheses formulated above, we conducted two experiments, each split into a Familiarization and a Main Part. The experiments were approved by the ethics committee of Paderborn University. The second experiment was preregistered.^1^ For both experiments, a 24″ monitor with a resolution of 1,920 × 1,080 px and a 60Hz frame rate was used, and stimuli were presented against a gray background (RGB: 192, 192, 192). In the first part, participants were familiarized with the stimulus material and could practice categorizing the forms. The Main Part of Experiment 1 is a single-factor within-subjects design. We manipulated the factor Comparison, which consisted of the three levels BlueBlue, RedRed, and BlueRed, determining which types of stimuli were used as probe and reference.

The main part of Experiment 2 follows a 2 × 3 within-subjects design. We manipulated the factors Classification Correctness and SAL-Option (present vs. absent). In each trial, two images with one classification were presented. Based on the Classification Correctness, the classification for each of these images could be correct for both, wrong for both, or correct for one image and wrong for the other. The factor SAL-Option determined whether participants could only respond that the classification was correct or not, or if they could also respond “show again later.” In 25% of the trials, the SAL-option was present. It was counterbalanced whether the SAL-option was present for Type A or Type B forms and whether it was present in the first or second half of the Main Part.

Furthermore, to conduct the TVA-TOJ analysis, we chose seven levels of stimulus-onset asynchrony (SOA). In Experiment 1, there were 20 trials per SOA level per Comparison, and in Experiment 2, we ensured at least 28 trials per SOA level per Classification Correctness, resulting in the trial frequencies that are summarized in Table 1. The image classifications were not AI-generated. The forms were classified so that they fit the design described above.

**Table 1:** Trials per SOA per condition.

				**SOA levels (ms)**				
		–100	–50	–16.6667	0	16.6667	50	100
*n* trials	Exp. 1	20	20	20	20	20	20	20
	Exp. 2	28	40	48	48	48	40	28

### 2.1 Participants

We recruited German-speaking students enrolled at Paderborn University via announcements during lectures and mailing lists. Participants had to be at least 18 years old. Persons with visual color deficiencies were excluded from participation. Participation was compensated with 10€ per hour or course credit, and the three best-performing participants were rewarded with a 20€ bonus. Participants in Experiment 1 (*N* = 32) were between 18 and 39 years old (*M* = 23.55, *SD* = 4.3). 79.41% identified as female. Participants in Experiment 2 (*N* = 51) were between 18 and 34 years old (*M* = 23.35, *SD* = 3.72) with 68.63% identifying as female. Participation in both experiments was allowed, and 35.29% of Experiment 2's sample also participated in Experiment 1. The TOJ performance of two participants in Experiment 2 was at chance level for all SOAs, indicating careless and insufficient effort in responding. These participants were excluded from all subsequent analyses.

### 2.2 Stimulus material

**Figure 1:**
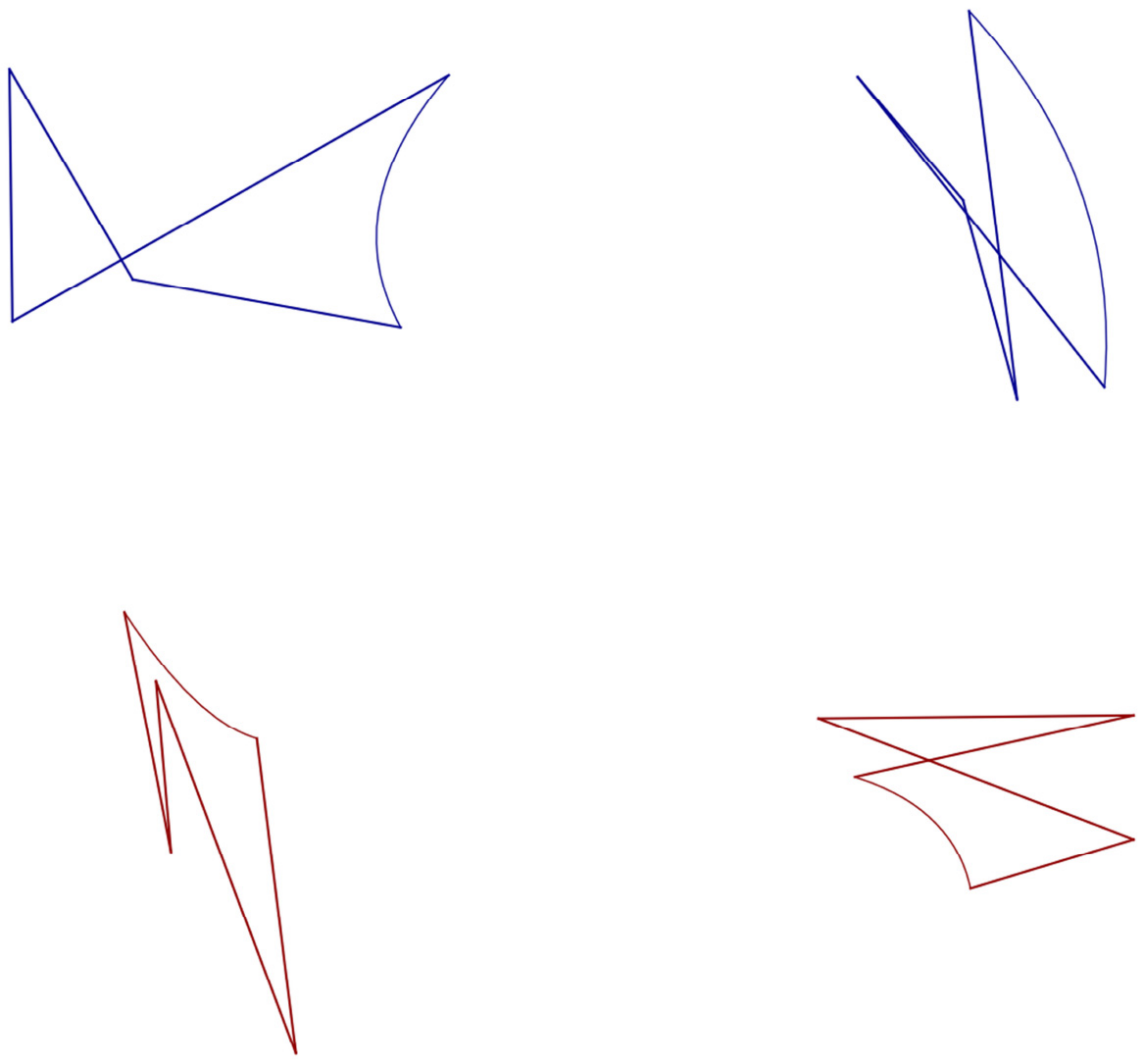
Examples of the used stimulus material (left: Type A, right: Type B).

The stimulus material was based on another study on erroneous image classifications (Peters and Scharlau, 2025). The stimuli are two-dimensional forms generated in Python with *matplotlib*. The stimuli of the present experiments are newly generated but share the same features as the stimuli used in Peters and Scharlau (2025). All forms have five points, five straight lines, and one curved line (for examples, see Figure 1).

The forms vary by three features: their color (blue, RGB: 0,0,139; red, RGB: 139,0,0), the curvature (convex vs. concave) of their curved line, and their width-to-height ratio. Depending on these features, we formulated the following categorization rules. The forms are categorized as Type A if they are either:

- blue, have a concave line, and are wider than high, or
- red, have a concave line, and are higher than wide.

They are categorized as Type B if they are either:

- blue, have a convex line, and are higher than wide, or
- red, have a concave line, and are wider than high.

In general, the blue forms are easier to categorize than the red. For the red forms, the closer the width-to-height ratio is to 1, the more difficult they are to categorize. This does not apply to the blue forms because if the categorization rules are fully understood, the width-to-height ratio is not necessary to categorize them.

### 2.3 Procedure—Experiment 1

#### 2.3.1 Familiarization

After giving their consent to the terms of the experiment, participants were informed that the following experiment is split into the Familiarization and the Main Part. Before the Familiarization Part, they received information about the stimuli and the features that were relevant for categorizing the material. They were informed that they should learn to categorize the stimuli into Type A and B and that the following procedure would be repeated once or, depending on their performance, twice. Participants went through 6 blocks of 4 trials with examples of correctly categorized forms, and 10 trials without examples. This procedure was repeated; this time, however, with 20 trials without examples. If the participants answered at least 80% of the trials correctly, they continued with the Main Part. Otherwise, the practice with and without examples was repeated once more, again with 20 trials without examples. Afterward, regardless of performance, all participants continued with the Main Part. For each repetition of the practice part, new stimuli were used. Participants received auditory feedback throughout all trials.

#### 2.3.2 Main Experiment

First, participants were instructed about the upcoming procedure verbally and on screen. They were instructed to try to respond as accurately as possible. They were reminded about the performance-based bonus. The participants were informed that the bonus would be based on both their TOJ performance and their performance in the query trials. With this incentivization, we wanted to ensure that participants focused on both tasks. Each trial consisted of a TOJ with two forms. In 25% of the trials, a query asking which type the two forms belong to followed. At the beginning of each trial, a fixation point at the center of the screen appeared, and two forms, one on each side, were presented with a 300px vertical offset. Unbeknownst to the participants, in the *BlueRed* and the *RedRed* comparisons, the probe stimulus was more difficult to categorize. In the BlueRed comparison, the probe was always the red form. In the *RedRed* comparison, the probe always had a width-to-height ratio more difficult to judge than that of the reference. In the *BlueBlue* comparison, both types of blue forms were equally often probe or reference, for which no difficulty difference was expected.

After a 700 ms fixation time, an offset-onset flicker occurred for the two stimuli. The delay between and the order of the probe and reference flicker was determined by the SOA values. For negative SOAs, the probe led; for positive SOAs, the reference led; and for the zero SOA, the flicker occurred simultaneously. Whether the probe was presented on the left and the reference on the right or vice versa was counterbalanced. The participants had to judge which of the two images flickered first by pressing “Q” for the left and “P” for the right stimulus.

If the TOJ was followed by the query about the forms, the participants had to judge to which type each form belonged. They had to respond within 2 s via key-press (“A” for Type A, “L” for Type B; the keys were marked with A and B on the keyboard).

### 2.4 Procedure—Experiment 2

The Familiarization Part followed the same procedure as in Experiment 1. As soon as participants reached at least 80% correct in a categorization block, the main part of Experiment 2 followed. At most, two repetitions of the Familiarization were offered. If the participant did not achieve 80% correct at least once, they could not participate in the Main Part. Before the Main Part, participants were informed about the upcoming procedure verbally and on screen. Participants were informed that they would not see actual AI classification, but should imagine that the presented classifications were AI-generated.^2^ To help participants imagine this, the classifications were labeled and referred to as AI advice throughout the remainder of the experiment.

At the beginning of the Main Part, participants had 48 tutorial trials to practice the task. In the tutorial, for each trial, participants were told on screen which classification of the mock-up AI had been generated for the upcoming two images, which remained visible throughout the whole trial. After one second, two images and a fixation mark appeared. After a brief delay (1,700–1,900 ms, randomized), an offset-onset flicker happened for the two stimuli in the same way as in Experiment 1. The fixation mark was either an “A” or a “B,” depending on the classification. After the tutorial, the classification was only indicated by the fixation mark, and each trial started with the appearance of the fixation mark and the two forms. In the *1each* condition, the probe stimulus was always the incorrect classification.

**Figure 2:**
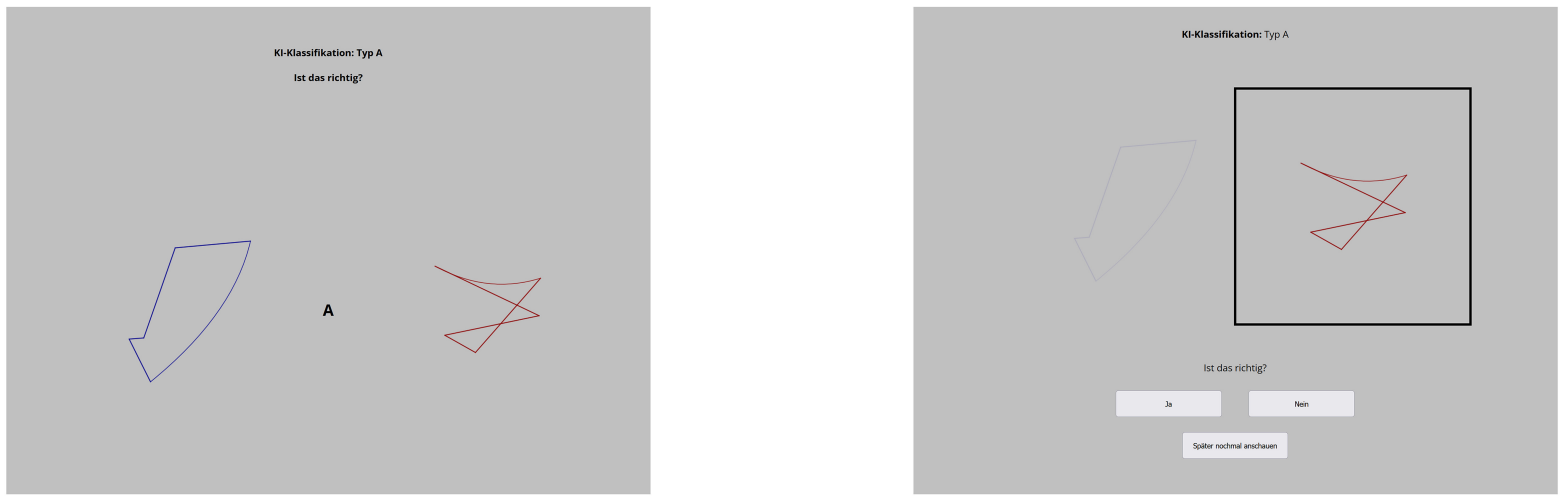
Setup of the main part of experiment 2 [**(left)** TOJ, **(right)** judgment about the AI classification with SAL-option].

After each TOJ, the participants were asked to judge via key-press whether the classification was correct (“A”) or false (“L”). When it was a SAL-option-present trial, they could also press “Space” to judge the image later. To visualize their options, boxes appeared on screen as shown in Figure 2. Participants judged these images one at a time. The image to be judged was visually highlighted, while the other one was grayed out. Both images had to be judged, and whether the left or right image was judged first was counterbalanced. The participants had 3 s to make each judgment. If they were too slow, the trial was recorded as no response, and a warning appeared to answer more quickly.

To reduce task demands for the participants, we chose to vary the image classification and the SAL-option not trial-wise but block-wise. Otherwise, the participants would have had to check which classification was made each trial, leading to a noisier measurement. Each block consisted of 30 trials. The classified type changed each block; whether the first block was Type A or B was counterbalanced across participants. For both classification types, the trial count for each level of Classification Correctness was equal across all blocks but was randomized within each block (across all blocks, the three levels of Classification Correctness occurred 280 times each, but in one block, one of the three levels could by chance occur, e.g., 12 times out of 20).

At the end of the experiment, participants had to judge if they focused more on forms where they were certain that the AI classification was correct or on the forms where they were uncertain that the classification was correct on a 5-point Likert scale. Furthermore, they were asked to approximate how often, on a scale from 0 to 100%, the AI classification was correct. Moreover, self-reported trust and distrust were assessed six times throughout the experiment via single items on a seven-point Likert scale (How much do you “trust”/“distrust” the AI classification?). This is beyond the scope of the article and is thus not included in the analysis.

### 2.5 Statistical analysis

For the statistical analysis, we used RStudio (R version 4.4.0), Jupyter Notebooks (Python version 3.12.10), and JASP (version 0.19.3, JASP Team, 2025). The Bayesian visual attention model has been implemented using *pymc5* (Abril-Pla et al., 2023) and applied to the data using the *NoUTurn Sampler* (Hoffman and Gelman, 2014, with 4 chains, 16,000 tuning samples, 24,000 draws). Further Bayesian tests were calculated in JASP.

#### 2.5.1 Bayesian TVA-TOJ model

For the TVA-based analysis, we used the TVA-TOJ model by Tünnermann et al. (2015). This model characterizes the probability of categorizing the probe as flickered first ($P_{\text{probe first}}$), dependent on the processing speed distributed on each stimulus (*v*_*p*(*robe*)_& *v*_*r*(*eference*)_) and the respective SOA. As briefly mentioned in Section 1.1, this approach allows us to estimate the attentional capacity *C* and the attentional weight *w*. Both are theoretically meaningful and easily interpretable parameters. *C* quantifies in Hertz (*Hz*) how many stimuli in the given setup are being processed. Thus, in the TVA-TOJ approach, *C* = 90*Hz* means that the flickering event of 90 stimuli can be processed within one second.^3^

*w* quantifies how much of this capacity a stimulus receives. Typically, the attentional weight for the probe stimulus is reported. A *w*_*probe*_ = 0.6 can be interpreted as the probe receiving 60% of the attentional capacity. Given that only two stimuli (probe and reference) are considered, the reference stimulus receives 40% of the attentional capacity. For simplicity, in the following, we will refer to *w*_*probe*_ as *w*.

To derive *C* and *w*, we use the observed frequencies with which the participants judged that the probe flickered first across the different SOA levels. For readers interested in the actual formalization of this, turn to the following paragraphs. Otherwise, it suffices to state that the more accurate the judgment of the temporal order of the probe and reference is, the higher the attentional capacity *C*. With regards to *w*, the more often the probe is judged first, the more attention the probe receives, which would be reflected in a *w* > 0.5.

This characterization is formally expressed in Equation 1 and can be subdivided into three cases.


$$\begin{array}{l} P_{\text{probe first}}(\theta |v_{p},v_{r},SOA)=\left\{ \begin{array}{l} \underset{\text{Case 1}}{\underset{︸}{1-e^{-v_{p}|SOA|}}}+\underset{Case2}{\underset{︸}{e^{-v_{p}|SOA|}(\frac{v_{p}}{v_{p}+v_{r}})}}\\ \;if\;SOA<0\\ \;\underset{Case3}{\underset{︸}{e^{-v_{r}|SOA|}(\frac{v_{p}}{v_{p}+v_{r}})}}\\ \;if\;SOA\geq 0 \end{array}\right. \end{array}$$ (1)

Case 1 applies if the SOA is negative (probe flickers before reference) and the processing of the probe flicker finishes before the reference's flickering starts its race for encoding. In this case, $P_{\text{probe first}}$ follows a cumulative exponential distribution according to TVA assumptions about encoding times. Case 2 applies if the SOA is negative, but the processing of the probe flicker does not finish before the reference flicker starts to race. Then, the probe's encoding probability is described by the complement of Case 1

Case 3 applies if the SOA is positive (reference flickers before probe). This case is analogous to Case 2, namely that the reference has not finished processing during the SOA (i.e., complementary probability to the one given by the distribution function of the exponential distribution), and thus probe and reference race together. The probe then wins the race with the probability of its relative processing speed advantage according to Luce choice rule. As a data model for $P_{\text{probe first}}$, we used a binomial distribution because it is the maximum entropy distribution for two-alternative choice data (McElreath, 2020).

So far, we have parameterized the TVA-TOJ model by *v*_*p*_ and *v*_*r*_ to be consistent with its derivation from TVA assumptions. Because we are interested in *C* and *w* for testing our hypotheses, a reparameterization in terms of *C* and *w* is needed. Following the TVA, we can substitute *v*_*p*_ and *v*_*r*_ according to (Equation 2; Bundesen, 1990) to directly estimate *C* and *w* from our model. When we substitute *v*_*p*_ and *v*_*r*_ in this way, we can estimate C and w from our model directly.


$$\begin{array}{l} v_{p}=C\cdot w\\ v_{r}=C\cdot (1-w) \end{array}$$ (2)

**Figure 3:**
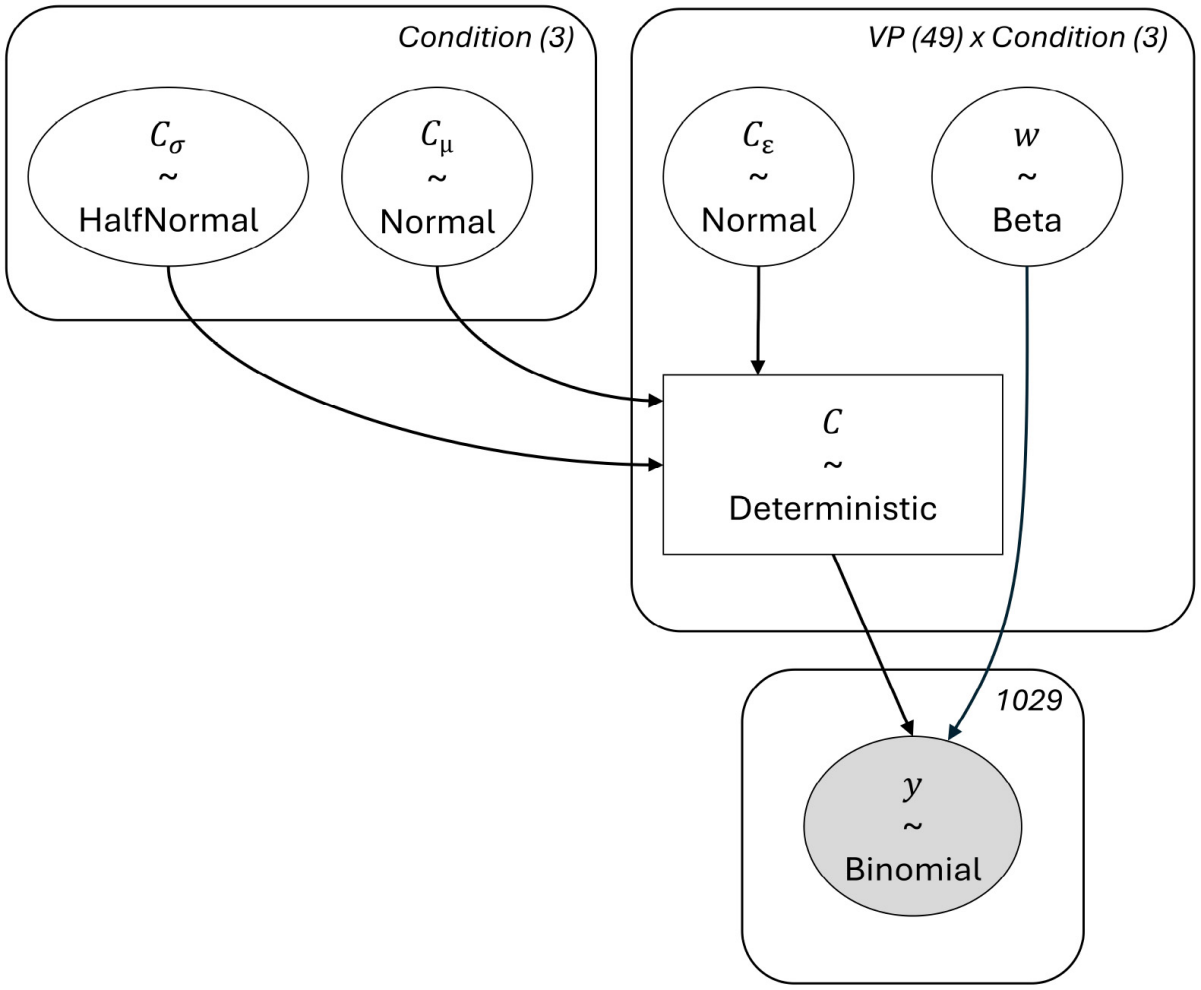
Structure of the Bayesian model for the TVA-TOJ analysis.

As we conducted a Bayesian estimation of the model's parameters, we also had to specify priors. We chose a hierarchical non-centered implementation because shrinkage to the group mean is known to improve estimates on average (McElreath, 2020). The hierarchical non-centered implementation of the TVA-TOJ model has been proposed by Tünnermann (2024) and has been applied in Biermeier et al. (2024); (Banh et al. (2024). The parameters of the hyperpriors (Equation 3) are chosen to match the empirical priors by (Tünnermann 2024). An overview of the described model is given in Figure 3. For the standard model diagnostics, including prior- and posterior-predictive simulations, please refer to the supplementary materials.


$$\begin{array}{l} C_{\mu }\;\sim Normal(\mu =-3,\sigma =1)\\ C_{\sigma }\;\sim HalfNormal(\sigma =0.5)\\ C_{e[i]}\sim Normal(\mu =0,\sigma =1)\;\forall i\in Participants\\ \;C_{i}\;=e^{C_{\mu }+C_{\sigma }*C_{e[i]}}\;\forall i\in Participants\\ \;w_{i}\;\sim Beta(1,1)\;\forall i\in Participants \end{array}$$ (3)

#### 2.5.2 Power Analysis

We conducted a Bayesian power search (Kruschke, 2014) by simulating data from and fitting the data to the TVA-TOJ model. The power search was implemented using the general workflow and simulation of Tünnermann (2024) along with the convenience wrappers and modeling facility of Biermeier (2025). Consider Algorithm 1 for details. The power search indicated that at least 43 participants are needed to recover all expected effects (i.e., the recovered difference excluded a minimal difference considered indistinguishable from zero) with a power of 0.83 [0.78, 0.88].

## 3 Results

### 3.1 Experiment 1

**Figure 4:**
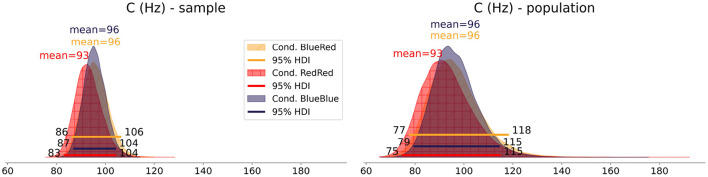
**(Left)** Mean attentional capacity *C* for each comparison type (sample estimates). **(Right)** Mean attentional capacity *C* for each comparison type (population estimates).

Figure 4 shows the *C* estimates of the three comparison types. We report the population estimate of C (*C*_μ_) and the C sample mean (average across individual *C* estimates). Both estimations result in the same distribution mean. As expected from a modeling point of view, C estimates on the sample level are more certain than on the population level; i.e., their highest density intervals (HDIs) are narrower. This phenomenon is rooted in our assumption about *C*_μ_. *C*_μ_ reflects the uncertainty that our sample is just one random sample and that other, more different samples may exist, while our sample-level *C* estimate does not. The *C* estimates are very similar to each other and indicate a high processing speed compared to other TVA assessments (e.g., Krüger et al., 2021; Espeseth et al., 2014).

**Figure 5:**
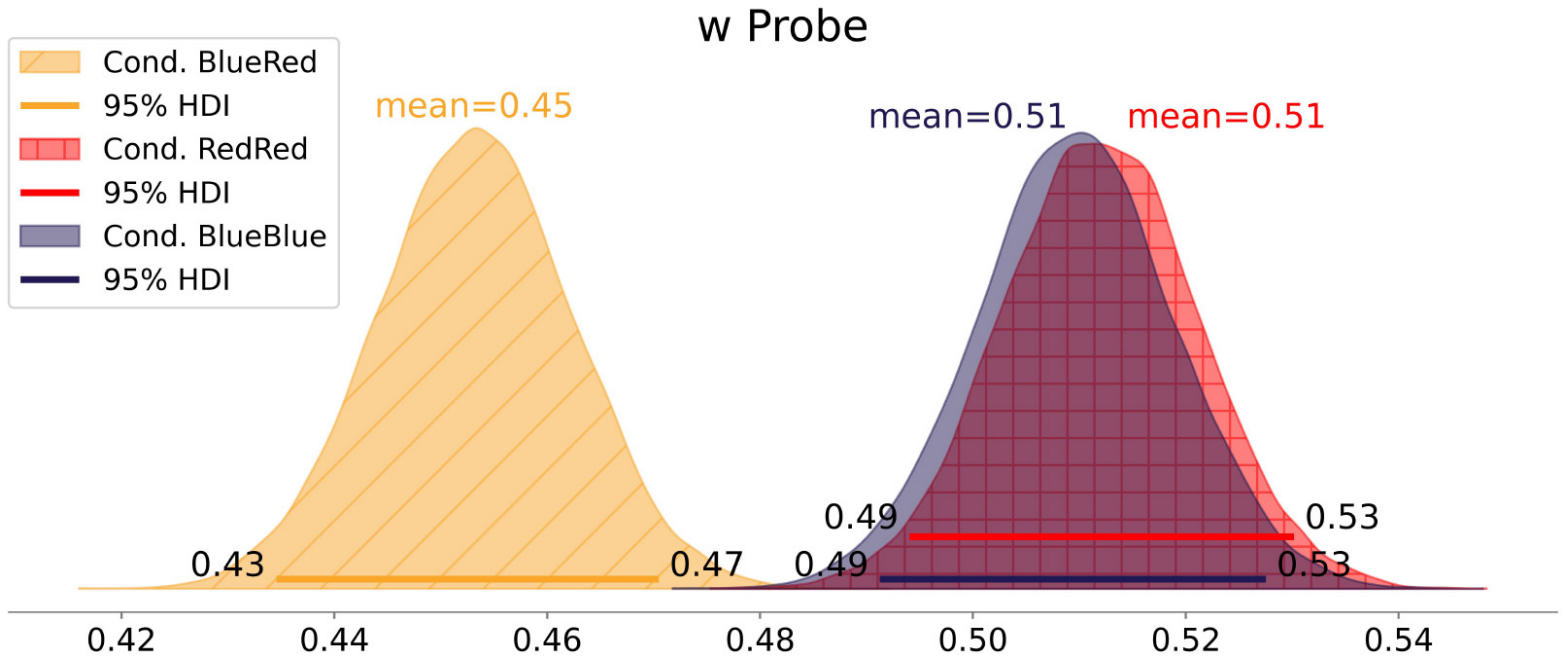
Experiment 1: mean attentional weight *w* of each condition.

In Figure 5, we report the *w* estimates of the three comparison types. In the *BlueBlue* and *RedRed* comparisons, *w* centers at 0.51 [0.49, 0.53] and 0.51 [0.49, 0.53], respectively. Contrary to our expectation, *w* in the *BlueRed* comparison is below 0.5 at 0.45 [0.44, 0.47]. This indicates that the easier stimuli received more attention than the more difficult ones.

Furthermore, we checked that the stimuli we considered more difficult based on previous studies were indeed more difficult in this sample as well. This is confirmed by the frequency of correct responses in Tables 2, 3.

**Table 2:** Contingency table for the frequencies of correct responses split by the color of the forms.

**Form color**	**Response correct**		**Total**
	**False**	**True**	
Blue	1, 212 (30.72%)	2, 734 (69.29%)	3, 946
Red	1, 474 (37.49%)	2, 458 (62.51%)	3, 932
**Total**	**2, 686** **(34.10%)**	**5, 192** **(65.91%)**	**7, 878**

Difference by color: *BF*_10_>10, 000.

**Table 3:** Contingency table for the frequencies of correct responses split by the forms' color and the difficulty of their width-to-height ratio.

**Width-to-height**			**Blue forms**			**Red forms**		
	**Response correct**		**Total**	**Response correct**		**Total**
	**False**	**True**		**False**	**True**	
Easy	587 (29.75%)	1, 386 (70.25%)	1, 973	636 (32.09%)	1, 346 (67.91%)	1, 982
Hard	625 (31.68%)	1, 348 (68.32%)	1, 973	8, 38 (42.97%)	1, 112 (57.03%)	1, 950
**Total**	**1, 212** **(30.72%)**	**2, 734** **(69.29%)**	**3, 946**	**1, 474** **(37.49%)**	**2, 458** **(62.51%)**	**3, 932**

Difference by width-to-height difficulty: blue forms: *BF*_10_ = 0.09; red forms: *BF*_10_>10, 000.

### 3.2 Experiment 2

**Figure 6:**
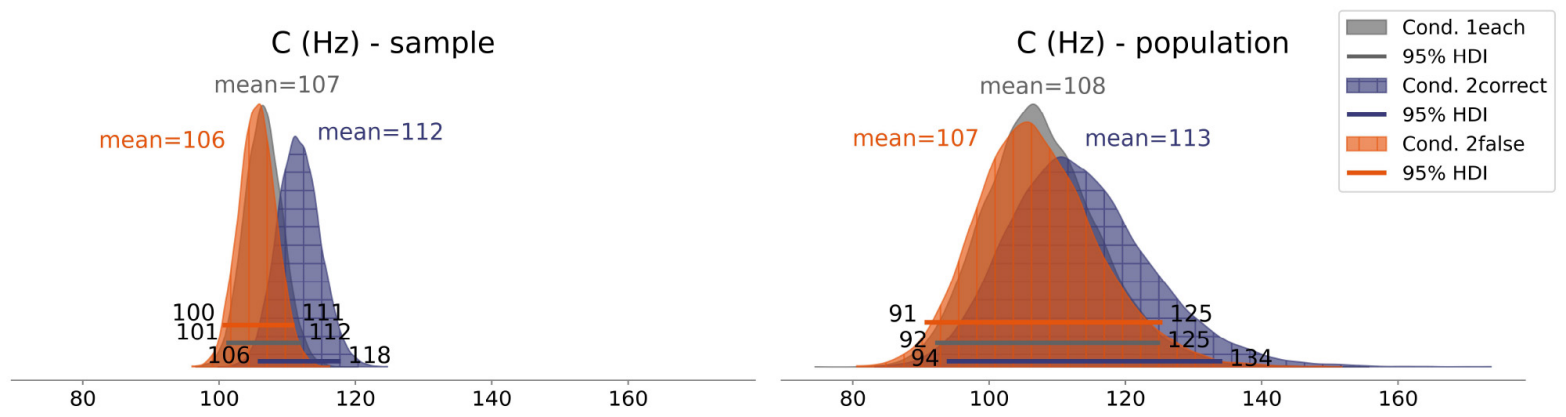
**(Left)** Mean attentional capacity *C* for each condition (sample estimates). **(Right)** Mean attentional capacity *C*_μ_ for each condition (population estimates).

**Figure 7:**
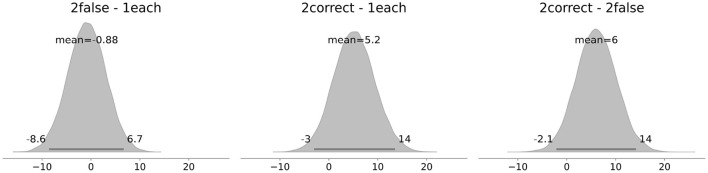
Differences in attentional capacity *C* between the conditions.

The attentional capacities *C*_μ_ of each condition are visualized in Figure 6. On average, the highest *C* is observed in the *2correct* Condition with a value of 112*Hz* [106, 118]. The values in the *2false* and *1each* conditions are very similar to each other, with a mean value of 106*Hz* [100, 111] and 107*Hz* [101, 112], respectively. To investigate *Hypothesis 2*, in Figure 7, the averaged individual *C* differences of the conditions are depicted. Comparing the *2false* to the *1each* condition, no meaningful *C* difference is observed, with zero well included in the HDI. Comparing the *2correct* to the *1each* condition, a tendency toward a positive difference is observed (5.2*Hz* [−3, 14]). Also, the posterior difference of the *2correct* and *2false* conditions (6*Hz* [−2.1, 14]) shows a tendency toward a positive difference, with 0 just included in the HDI. This indicates a smaller attentional capacity when both images are incorrectly classified. Comparing these differences and their HDIs to a region of practical equivalence (ROPE), set at −∞ to 5*Hz*, it is 1.49 times more likely that the 2correct-2false difference is above rather than within the ROPE. This can be interpreted as anecdotal evidence (Lee and Wagenmakers, 2014) for a positive difference and thus as weak support for *Hypothesis 2*. Taken together, as soon as one image is incorrectly classified, the attentional capacity *C* tends to decrease.

**Figure 8:**
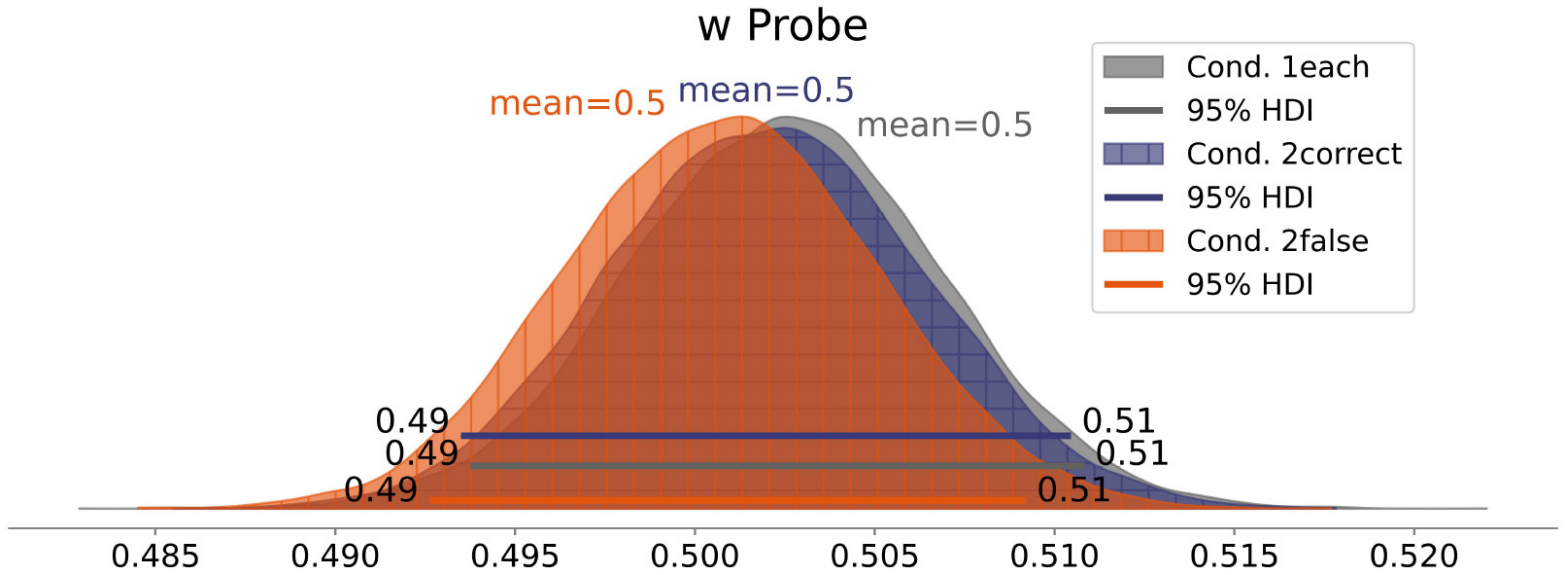
Mean attentional weight *w* for each condition.

**Figure 9:**
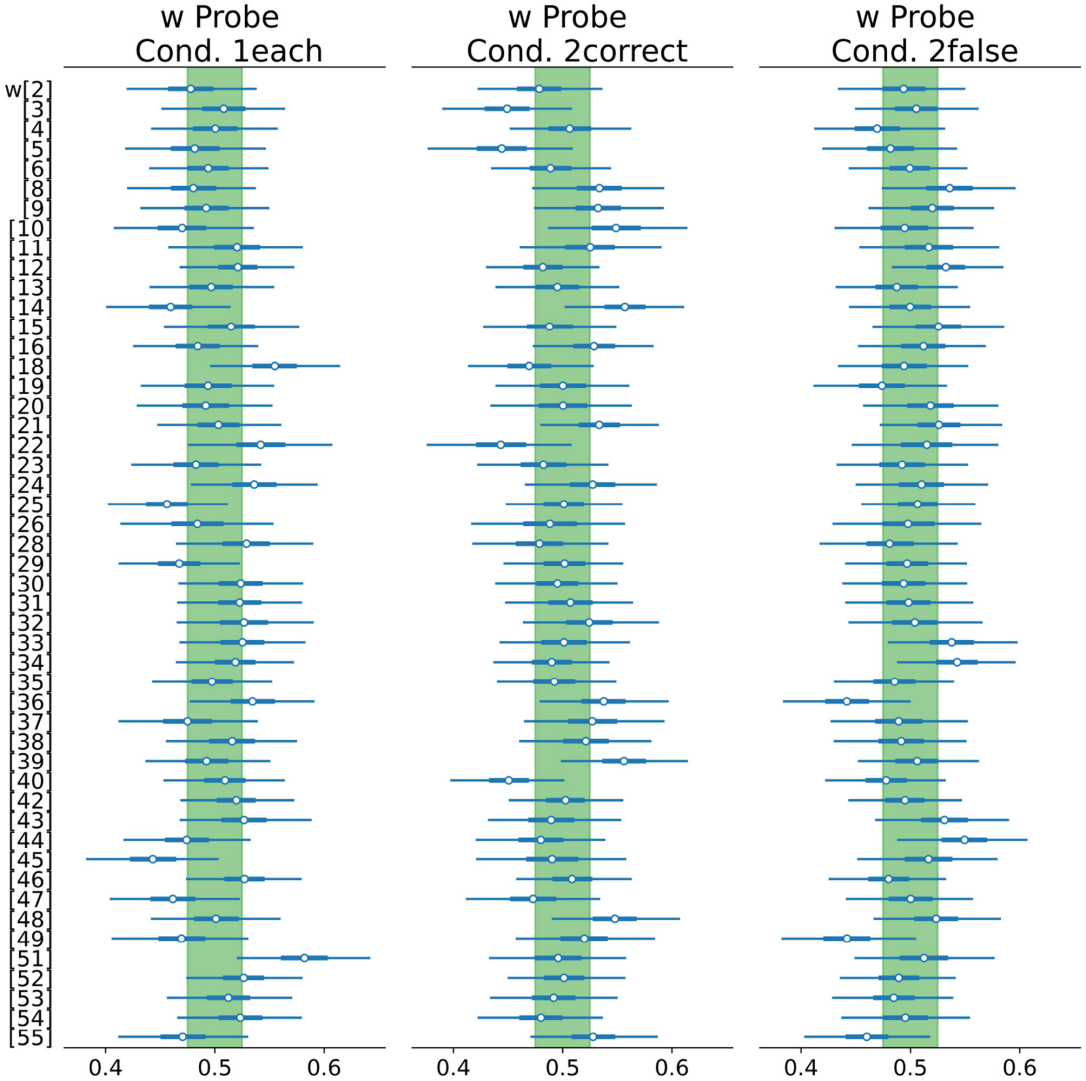
Forest plot of the individual attentional weights *w* split by condition. Green area: ROPE from 0.475 to 0.525.

Contrary to our expectation of *Hypothesis 3*, the posterior distribution of the attentional weight *w* of Condition *1each* (Figure 8) centers at 0.5 [0.49, 0.51], indicating no effect of Classification Correctness on the attentional weights' distribution. Inspecting the attentional weights of each participant (Figure 9), we can see both increases and decreases in the attentional weight of the probe stimulus (incorrect classification).

In terms of the SAL-option, we cannot draw any conclusions. Unfortunately, only 12 participants used this option at all, and if they did, they did so only a few times (*M* = 9.92, with a range of 1 to 56 times out of 420). Further investigation, with an adapted design that incentivizes the SAL-option more strongly, is needed to study *Hypothesis 4*.

### 3.3 Additional analyses

For further exploration of the observed effects, we performed additional analyses beyond what we had specified in our pre-registration. Firstly, we further investigated the individual *w* values. Based on the self-report at the end of the study, we grouped the participants. Even with the grouping, no pattern in the results is present (see Appendix for further details). What we do observe here is a difference in C depending on the post-questionnaire grouping. Across all three conditions, we observe that participants who reported that they focused more on the stimuli they were sure about had a lower attentional capacity C than the participants who reported focusing more on the stimuli they were unsure about.

Secondly, we tested for a possible relation between the *C* difference between the *2correct* and *2false* conditions and the participants' performance in judging the classification but did not observe any meaningful relationship (*r* = 0.04, *BF*_10_ = 0.18).

**Figure 10:**
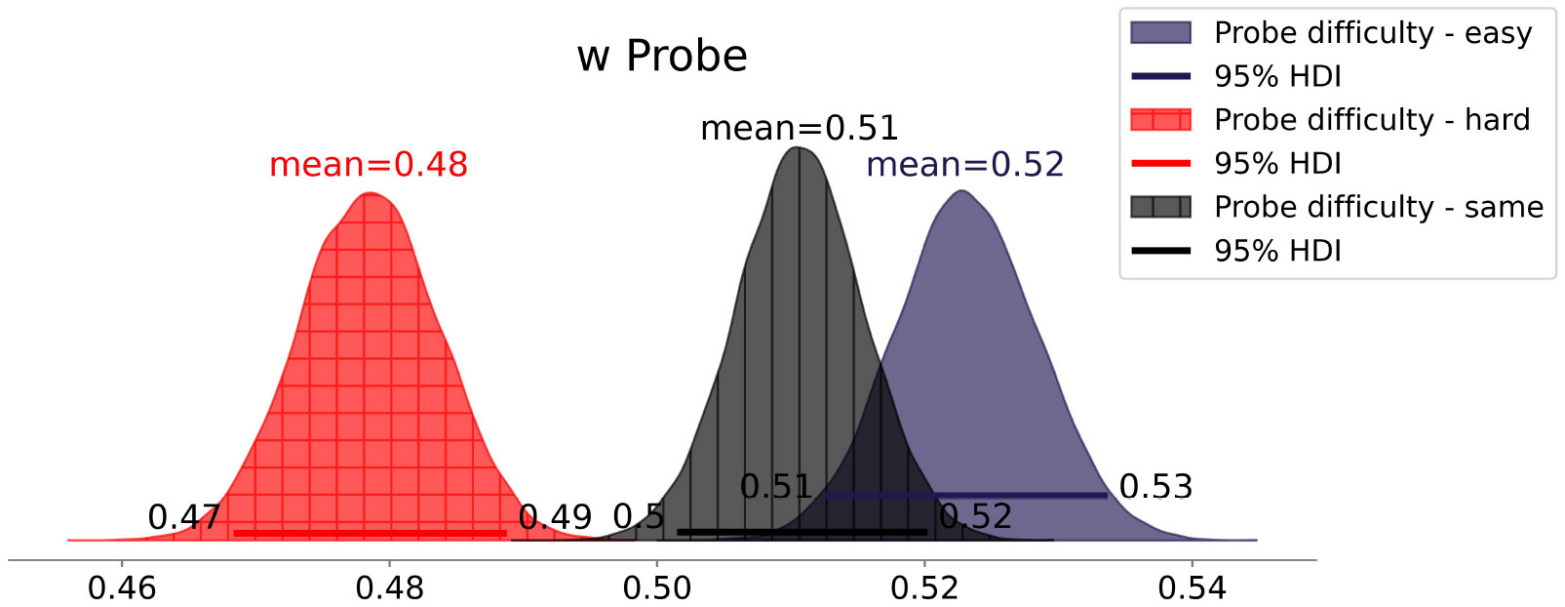
Mean attentional weight *w* split by the difficulty of the probe stimulus compared to the reference stimulus.

Furthermore, to corroborate the findings of Experiment 1, we also tested the effect of stimulus difficulty in Experiment 2. We conducted another TVA-TOJ analysis, now with the data split by stimulus difficulty. The probe was more difficult when it was red and the reference was blue, or, in the case both stimuli were red, when the probe's width-to-height ratio was more difficult to judge than the reference's ratio. The results are visualized in Figure 10. Again, the stimuli that were easier to categorize received more attention.

## 4 Discussion

In this contribution, we offer the perspective of visual attention as another aspect to investigate human-AI interaction. Appropriate trust and similar concepts have the complex aim of fostering users' abilities to notice errors and also to foster their intuition regarding when to expect errors. We have argued that the established measurements of self-reported (dis)trust and reliance are relevant but not sufficient to assess this range of consequences, which is why we suggested attention as an additional indicator. To test this potential indicator, we investigated visual attention toward correct and incorrect image classifications.

In Experiment 1, we first tested if the difficulty of categorizing the stimuli influences the participants' attentional capacity *C* or its distribution. Our results indicate that categorization difficulty does not affect the attentional capacity *C*. The capacity's distribution, assessed via the attentional weight *w*, is affected by the difficulty. Stimuli that were easier to categorize received more attention than those that were more difficult. An additional analysis with data from Experiment 2 further corroborates this finding. Overall, this contradicts *Hypothesis 1* and opposes our expectation, namely that more easily classified stimuli receive more attention.

The direction of the observed effect can, of course, be specific to the task setup. Especially, the time constraint for making the decisions that followed the TOJ task may have led to the increased attention toward the easier stimuli. Without this time constraint, the effect may reverse or disappear. However, the query response was sped up to ensure that participants would not separate the two tasks, and thus, removing the time constraint could alter the link between classification correctness and the attentional estimates. Alternatively, further research could test different forms of incentivization or allow participants to complete the TOJ either after the query or before and after the query.

In Experiment 2, participants encountered supposed AI image (mis)classifications. The results do not support *Hypothesis 3* because the Classification Correctness did not affect *w*. Even for the participants who reported that they focused on the stimuli they were not sure were being classified correctly, we only observed a small tendency of increased attention toward misclassified images. Overall, the small deviations of individual *w*s from 0.5 (i.e., uniform distribution of attention) and thereby the small expected differences in *w* are most probably caused by measurement uncertainty. Remember that the full posterior still assigns much probability to values close to 0.5.

In terms of the overall attentional capacity *C*, we observed a difference due to the Classification Correctness, supporting *Hypothesis 2*. When both stimuli were correctly classified, a higher attentional capacity was observed. The fact that this difference occurred as soon as one image was incorrectly classified indicates that there may be additional demands from the presence of misclassifications. This could mean that participants quickly noticed the misclassifications and prepared for the potentially more demanding response that the classification was incorrect. However, this is not supported by the second additional analysis, which did not show a relation between the C differences and the subsequent performance.

The fact that we observed a difference in the *C* estimates depending on the grouping for the additional analysis of *w* is interesting. Nevertheless, given the *post-hoc* nature of this analysis, we can only speculate about the causalities. It could be a motivational effect that leads to higher C values and also reporting that one focused on the forms they were unsure about. It could also be that participants who were struggling more with the TOJs could only focus on the stimuli they were sure about, which is reflected in their self-report, and those who performed the TOJ with more ease could allow themselves to focus on stimuli they were unsure about.

In summary, the participants' visual attention, estimated via the TVA-TOJ approach, is affected by our experimental manipulation, but neither of the two investigated parameters clearly indicates healthy distrust. The reduction of the attentional capacity indicates a slower processing speed for these images when image misclassifications were encountered. However, there was no relationship to the performance in the subsequent judgment task about these images. Therefore, it remains inconclusive if the reduced attentional capacity is otherwise beneficial. Further research that replicates this result pattern and tests for beneficial effects is needed. If such results could be established, only then could the attentional capacity serve as an indicator of healthy distrust.

The weight parameter *w* does not qualify as an indicator of healthy distrust in our scenario. We only observed a meaningful change in *w* because of the categorization difficulty, but not because of the Classification Correctness. This shows that the attentional weight is sensitive to participants' difficulty in categorizing the stimuli, but that it is not sensitive to the AI's correctness. Future research could investigate a systematic variation of the AI's correctness. Instead of having errors for all types of forms, the classifications could err for only a certain subtype of the stimuli. After allowing the participants to apprehend the systematic error, it would be interesting to test if this influences their attention toward the error-prone stimuli.

### 4.1 Limitations

A first limitation is that Experiment 2's sample only consists of subjects who performed well in categorizing the stimuli. While this was needed to ensure that participants could distinguish correct from incorrect classifications, it introduces a selection bias to our sample that may have influenced our results, thus preventing their generalizability. Further insights would be provided by using different performance requirements for participation and comparing the results.

Secondly, as mentioned in Section 2.4, our participants were informed about the mock-up nature of the AI advice. It certainly makes a difference, especially for self-reported trust and distrust, to interact with actual AI instead of only imagining that one receives AI advice. However, for our purpose of testing whether the correctness of a classification influences visual attention, we regard this difference as less relevant. We assumed that the effects of noticing incorrectly classified stimuli would be at least similar, regardless of whether participants knew about the mock-up nature of the AI advice. Nonetheless, if extending our approach to a naive sample, it is preferable to instruct participants that they will interact with actual AI.

The observed change in *w* due to the categorization difficulty was mainly observed when comparing the easier blue forms to the more difficult red forms. Therefore, instead of the difficulty, the color of the forms could also provide a simpler explanation for the results. Indeed, the luminance difference (L values in CIELab) between the blue color and the background color is larger than the difference between the red color and the background, which can lead to a higher saliency and the observed change in attentional weighting (Wolfe and Horowitz, 2004; Krüger et al., 2016). However, the luminance differences are of similar magnitude, and the lines were rather thin compared to colored circles or thick bars, with which such saliency effects are typically investigated. As the participants were engaged in the more abstract and higher-level task of categorizing the forms, it is unlikely that the observed effect stems solely from the colors' saliency. Nonetheless, switching the colors or using different colors would further inform the present results.

Moreover, as one reviewer pointed out, this would allow for a more nuanced analysis of what exactly drives the observed change in *w*. In the TVA, *w* is defined as the multiplication of sensory evidence, i.e., how easily the stimuli's features are encoded, and pertinence, i.e., how much importance is given to each of these features. The former is influenced, for example, by the discussed luminance difference. The latter is influenced, for example, by the categorization rules, because if one feature is more relevant than another for a participant's decision to categorize the stimuli, this feature has a higher pertinence. Suppose a change in sensory evidence, such as switching colors, does not change the observed effect. In that case, we could attribute the effect with more certainty to the pertinence. To fully disentangle whether the *w* effect stems from the sensory evidence or the pertinence, it would be best to keep the sensory evidence constant and only manipulate the pertinence. Ideally, two sets of categorization rules are needed that vary in the importance of certain aspects of the stimuli and still apply to the same stimuli. This is not possible with the present stimulus set.

Furthermore, in comparison to other TVA studies (Krüger et al., 2021; Espeseth et al., 2014), our *C* estimates are high. Potential reasons are that most of our participants were familiar with TOJ tasks, that we did not use any distractors during the TOJs, and that our stimuli were comparably large, which was needed to ensure that the forms' type could be identified. Moreover, the combination of TOJs and the queries about the forms may have made the experiment less monotonous than a pure TOJ paradigm. All of this can contribute to a simpler or less error-prone TOJ and thus to higher *C* values. Given our within-subjects design, this is unproblematic for our comparison of conditions, but it leads to more uncertainty about the *C* estimates.

As recently shown (Banh and Scharlau, 2025), the higher the estimated *C* values are, the larger their HDI width is, and thus the uncertainty about them. High *C* values are obtained when participants make very few errors during the TOJs. For high *C* values, a single error leads to a much larger change in *C* than it does for lower *C* values. Therefore, future studies should ensure a certain difficulty of their TOJs to obtain sharper estimations of *C*. Changes to the previously mentioned issues of stimulus and task design would ensure that.

## 5 Conclusion

To sum up, the usefulness of our attempt to assess visual attention as an indicator for healthy distrust, appropriate trust, or similar notions is limited. While we do observe changes due to the Classification Correctness, only the capacity is affected. For this, the causal interpretation is difficult. In terms of *w*, the Classification Correctness did not have an effect. The fact that the categorization difficulty influenced *w* in both experiments shows that this formal assessment of the weighting of visual attention is sensitive to such a task, which is why we, as described above, advocate further empirical exploration.

In general, research on human-AI interaction needs to continue investigating (dis)trust and reliance, and it should combine this with further analysis to fully address the complexity of appropriate trust and healthy distrust. For instance, relevant conceptual insights are provided by interpersonal research on epistemic vigilance and its application to the domain of developmental psychology (Mercier, 2017; Sperber et al., 2010; Koenig and Harris, 2007). To improve the assessment of knowing when to doubt, the core contribution of the notion of healthy distrust is an important endeavor. Some approaches on decision time or mouse tracking can also be useful additions (Dechant et al., 2023; Vereschak et al., 2021). However, in comparison to our approach, these measurements lack the formal definition and theoretical base that the TVA provides.

Regardless of the exact approach, the recent developments of AI applications, most prominently in the interaction with LLM-based applications, emphasize the need to foster critical usage; for that, we need ways to assess and evaluate healthy distrust. Due to the complexity of how such healthy distrust can manifest itself, it is most likely that a combination of measurement types is needed to fully cover this important and complex desideratum. We hope our contribution makes this complexity more apparent and stimulates further research on it.

## Figures and Tables

**Figure A1:**
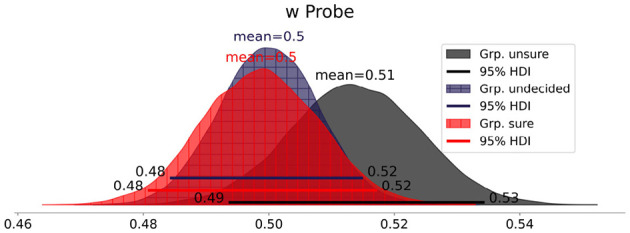
*w* Probe estimates of condition 1each after applying the *post-hoc* grouping.

**Figure A2:**
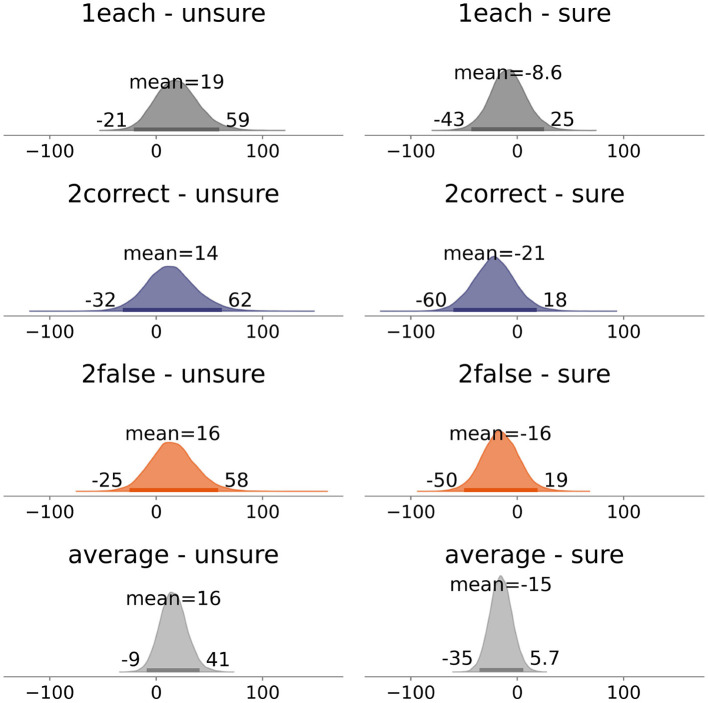
Portion of *C* (Hz) that is explained by the *post-hoc* grouping. Reported are the values of group unsure and group sure, with group undecided serving as baseline.

## Data Availability

The datasets presented in this study can be found in online repositories. The names of the repository/repositories and accession number(s) can be found at: OSF repository: https://osf.io/yhp9r.

## Footnotes

1 For the preregistration, see https://doi.org/10.17605/OSF.IO/NZKX9.

2 We chose this instruction, because we recruited from a pool of potential subjects that already participated in studies where they were deceived about mock-up AI advice being actual AI advice. Therefore, they were unlikely to be convinced again.

3 It should be noted that still, the visual short-term memory limits the amount of stimuli that can be represented in parallel, which is defined in the TVA as the parameter *K*.

## Appendix

### Power analysis

#### Assumptions

1. *C*_*Condition*0_~*Normal*(μ = 50*Hz*, σ = 20*Hz*)
2. *C*_*Condition*1_~*Normal*(μ = 60*Hz*, σ = 20*Hz*)
3. *C*_*Condition*2_~*Normal*(μ = 50*Hz*, σ = 20*Hz*)
4. *w*_*Condition*0_~*Beta*(α = 50, β = 50)
5. *w*_*Condition*1_~*Beta*(α = 50, β = 50)
6. *w*_*Condition*2_~*Beta*(α = 60, β = 40)

#### Success Criteria

1. *mean*(Ĉ_Condition 1_ - Ĉ_Condition 0_) >3*Hz*
2. 95%−*HDI*_*lower*_(ŵ_Condition 2_) >0.525

### Additional analysis - post questionnaire grouping

The participants were sorted into three groups based on their self-report of whether they focused more on the stimuli that they were sure about that they were correctly classified, unsure about, or if they were undecided. After grouping the participants according to their self-reported focus on the stimuli, the *w* estimates indicate uniform distribution of attention to probe and reference or a slight preference for probe in Grp. unsure (*w* = 0.51). Thus, at most only in this group of participants, a slight tendency in the expected direction (Hypothesis 3) is observed.

During this additional analysis, we also inspected the C estimates. As one would expect, the overall *C* estimates remained unchanged. Interestingly, the grouping explains a portion of the variation of *C* estimates. Figure A2 visualizes the portion of *C* that is explained by the *post-hoc* grouping for Group unsure and Group sure compared to the Group undecided. From this, we can see that on average across the three conditions, participants who reported focusing more on the stimuli they were sure about had a lower attentional capacity *C* (−22*Hz* [−41, −2.1]). For participants who reported focusing more on the stimuli they were unsure about, a reverse but less convincing pattern is visible (9.7*Hz* [−13, 33]).

Algorithm 1 Bayesian power search.
